# Supplementary material for: 9-fold symmetry is not essential for centriole elongation and formation of new centriole-like structures
Source: Nat Commun. 2024 May 25;15:4467. doi: 10.1038/s41467-024-48831-y (PMC11127918; doi:10.1038/s41467-024-48831-y)
Supplement: Supplementary file 1 — Supplementary Information [file 41467_2024_48831_MOESM1_ESM.pdf]

Supplementary Information File

9-fold symmetry is not essential for centriole elongation and formation of new centriole-like structures

Pallavi Panda, Mark S. Ladinsky, and David M. Glover*

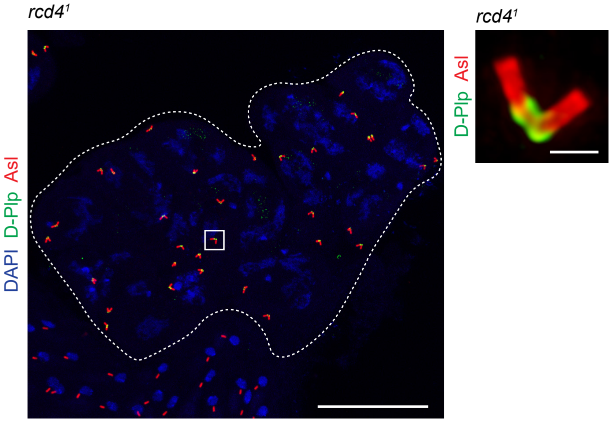

Supplementary Fig. 1: *rcd4¹* primary spermatocytes have two pairs of structurally normal centrioles. *rcd4¹* mature primary spermatocyte cyst immuno-stained to reveal elongated centrioles; Asl (red), D-Plp (green), and DNA (blue). Boundary of the 16-cell cyst is outlined with dotted-lines. Scale bar, 30 μm. Magnified example of an elongated centriole in a mature primary spermatocyte immuno-stained to reveal Asl (red) and D-Plp (green). Scale bar, 1μm. Experiment was repeated more than three times with similar results.

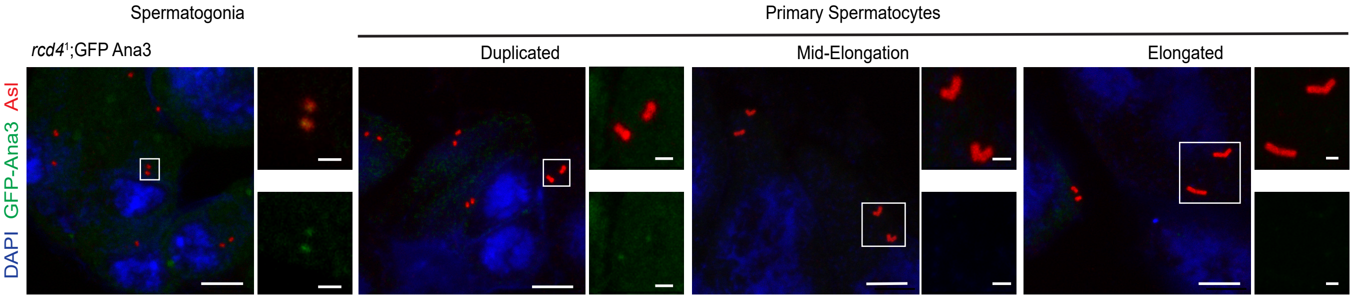

Supplementary Fig. 2: *rcd4¹* mutant spermatocyte centrioles loose Ana3 upon centriole elongation. *rcd4¹*;GFP-Ana3 spermatogonia and primary spermatocytes at centriole duplication, mid-elongation and elongated stages, stained to reveal GFP-Ana3 (green), Asl (red) and DNA (blue). Scale bars, 5μm and 1μm (insets). Experiment was repeated more than three times with similar results.

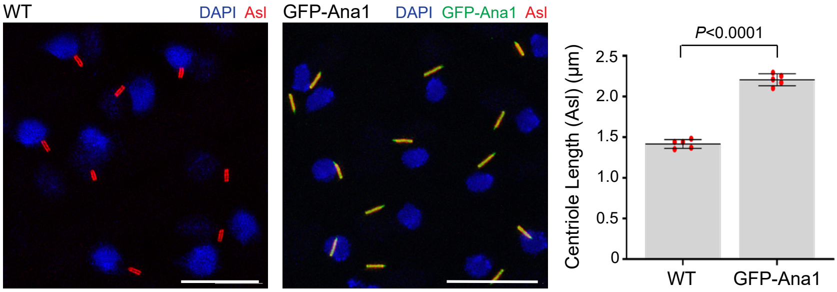

Supplementary Fig. 3: Overexpression of Ana1 results in longer centrioles. WT and poly-Ubiquitin driven Ana1 overexpression – single copy (GFP-Ana1) round spermatids were immuno-stained to reveal GFP-Ana1 (green), Asl (red), and DNA (blue). Scale bar, 10µm. Centriole length measurements were performed using Asl as a marker for centrioles in round spermatids. A two-tailed, unpaired t test was performed with a P-value of $P<0.0001$. Mean values for centriole length in WT and GFP-Ana1 round spermatids are 1.4µm and 2.2 µm, respectively. Mean values and SDs are shown for five independent testes per genotype. N= 184 round spermatids analyzed for WT and 176 round spermatids for GFP-Ana1 with n=~30 spermatids analyzed per testis. Source data are provided as a Source Data file. Experiment was repeated two times with similar results.

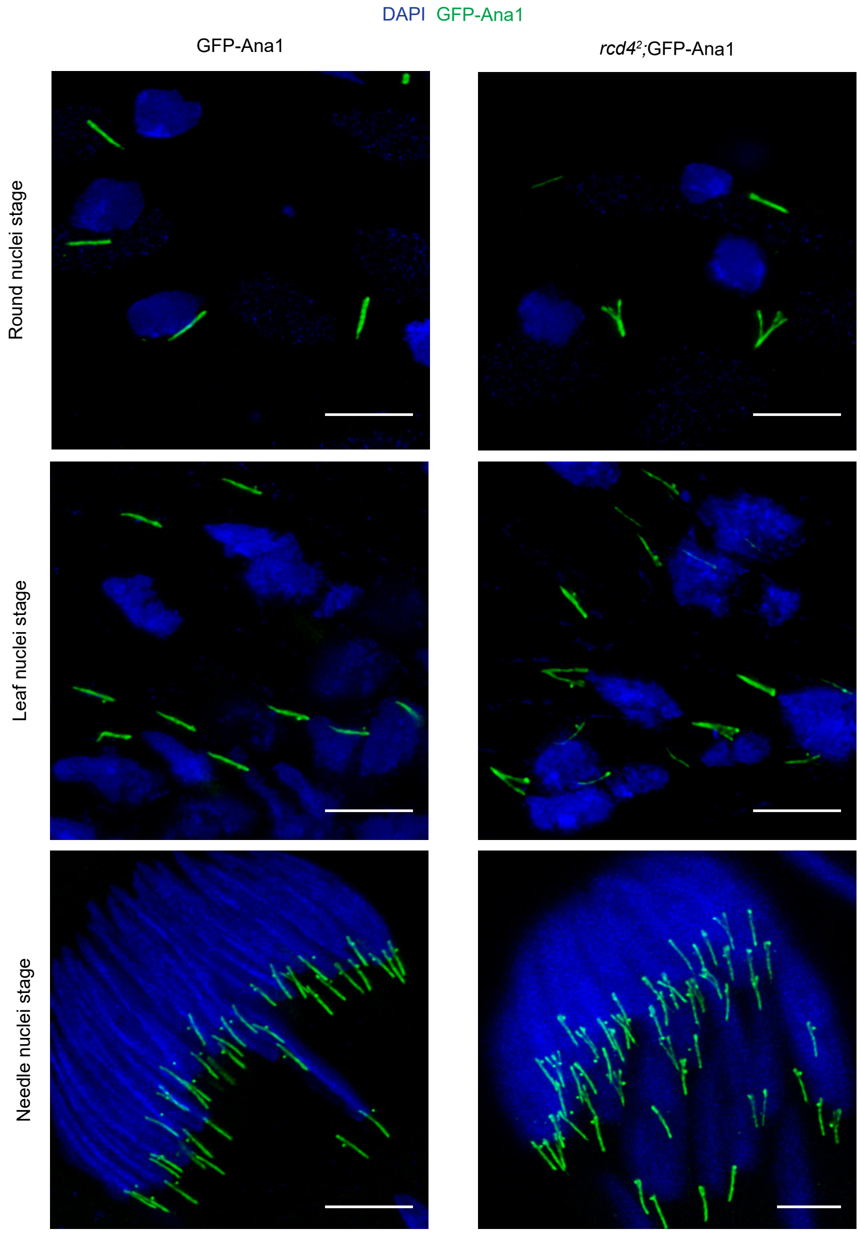

DAPI GFP-Ana1
GFP-Ana1
rcd4²;GFP-Ana1
Round nuclei stage
Leaf nuclei stage
Needle nuclei stage

Supplementary Fig. 4: Stages of spermatid elongation and their relationship to PCL formation. GFP-Ana1 and *rcd4*^{2}; GFP-Ana1 spermatids at round-, leaf-, and needle-like nuclear stages reveal GFP Ana1 (green) and DNA (blue). Scale bar, 5μm. Experiment was repeated three times with similar results.

Supplementary Movie Figure Legends

Supplementary Movie 1: Electron tomography of a centriole pair from WT primary spermatocyte. Video compilation of entire z-stack volume, including a three-dimensional model. Scale bar, 100nm.

Supplementary Movie 2: Electron tomography of a paired skinny centriole from *rcd4*^{2} primary spermatocyte. Video compilation of entire z-stack volume, including a three-dimensional model. Scale bar, 100nm.

Supplementary Movie 3: Electron tomography of a WT spermatid centriole. Video compilation of entire z-stack volume, including a three-dimensional model. Scale bar, 100nm.

Supplementary Movie 4: Electron tomography of a *rcd4*^{2};GFP-Ana1 spermatid centriole showing restricted splaying. Video compilation of entire z-stack volume. Scale bar, 200nm.

Supplementary Movie 5: Magnified electron tomography of a *rcd4*^{2};GFP-Ana1 spermatid centriole in Supplementary Video 4. Video compilation of entire z-stack volume, including a three-dimensional model. Scale bar, 50nm.

Supplementary Movie 6: Electron tomography of a splayed centriole in *rcd4*^{2};GFP-Ana1 spermatid. Video compilation of entire z-stack volume, including a three-dimensional model. Scale bar, 100nm.

Supplementary Table 1. Fly stocks used in the study

No.	Detailed genotype	Name in Text	Description	Source
1.	*w; rcd4^1/CyO, Tb, Roi*	*rcd4^1*	Chr II	[1]
2.	*w; rcd4^2/CyO, Tb, Roi*	*rcd4^2*	Chr II	[1]
3.	*w; Ubq-GFP-Ana3/TM6b*	-	Chr III	[1]
4.	*w; Ubq-Sas6-GFP/CyO*	-	Chr II	Glover Lab
5.	*w; Ubq-GFP-Gorab/TM6b*	-	Chr III	[2]
6.	*w; Ubq Sas4-RFP/TM6b*	-	Chr III	Glover Lab
7.	*w; Ubq GFP Ana1/CyO*	-	Chr III	Glover Lab
8.	*w; Ubq RFP Ana1/TM6C*	-	Chr II	Glover Lab

Supplementary References:

1. Panda, P. *et al.* Tissue specific requirement of Drosophila Rcd4 for centriole duplication and ciliogenesis. *Journal of Cell Biology* **219**, (2020).

2. Kovacs, L. *et al.* Gorab is a Golgi protein required for structure and duplication of Drosophila centrioles. *Nat Genet* **50**, 1021–1031 (2018).
