# Supplementary material for: 9-fold symmetry is not essential for centriole elongation and formation of new centriole-like structures
Source: Nat Commun. 2024 May 25;15:4467. doi: 10.1038/s41467-024-48831-y (PMC11127918; doi:10.1038/s41467-024-48831-y)
Supplement: Supplementary file 3 — Description of Additional Supplementary Files [file 41467_2024_48831_MOESM3_ESM.pdf]

Description of Additional Supplementary Files:

Supplementary Movie 1: Electron tomography of a centriole pair from WT primary spermatocyte. Video compilation of entire z-stack volume, including a three-dimensional model. Scale bar, 100nm.

Supplementary Movie 2: Electron tomography of a paired skinny centriole from rcd42 primary spermatocyte. Video compilation of entire z-stack volume, including a three-dimensional model. Scale bar, 100nm.

Supplementary Movie 3: Electron tomography of a WT spermatid centriole. Video compilation of entire z-stack volume, including a three-dimensional model. Scale bar, 100nm.

Supplementary Movie 4: Electron tomography of a rcd42 ;GFP-Ana1 spermatid centriole showing restricted splaying. Video compilation of entire z-stack volume. Scale bar, 200nm.

Supplementary Movie 5: Magnified electron tomography of a rcd42 ;GFP-Ana1 spermatid centriole in Supplementary Video 4. Video compilation of entire z-stack volume, including a three-dimensional model. Scale bar, 50nm.

Supplementary Movie 6: Electron tomography of a splayed centriole in rcd42 ;GFP-Ana1 spermatid. Video compilation of entire z-stack volume, including a three-dimensional model. Scale bar, 100nm.
