# Supplementary material for: 9-fold symmetry is not essential for centriole elongation and formation of new centriole-like structures
Source: Nat Commun. 2024 May 25;15:4467. doi: 10.1038/s41467-024-48831-y (PMC11127918; doi:10.1038/s41467-024-48831-y)
Supplement: Supplementary file 10 — Reporting Summary [file 41467_2024_48831_MOESM10_ESM.pdf]

nature portfolio

Corresponding author(s): David M Glover

Last updated by author(s): Apr 30, 2024

Reporting Summary

Nature Portfolio wishes to improve the reproducibility of the work that we publish. This form provides structure for consistency and transparency in reporting. For further information on Nature Portfolio policies, see our Editorial Policies and the Editorial Policy Checklist.

Statistics

For all statistical analyses, confirm that the following items are present in the figure legend, table legend, main text, or Methods section.

n/a	Confirmed	
☐	☒	The exact sample size (*n*) for each experimental group/condition, given as a discrete number and unit of measurement
☐	☒	A statement on whether measurements were taken from distinct samples or whether the same sample was measured repeatedly
☐	☒	The statistical test(s) used AND whether they are one- or two-sided<br>*Only common tests should be described solely by name; describe more complex techniques in the Methods section.*
☒	☐	A description of all covariates tested
☒	☐	A description of any assumptions or corrections, such as tests of normality and adjustment for multiple comparisons
☐	☒	A full description of the statistical parameters including central tendency (e.g. means) or other basic estimates (e.g. regression coefficient) AND variation (e.g. standard deviation) or associated estimates of uncertainty (e.g. confidence intervals)
☐	☒	For null hypothesis testing, the test statistic (e.g. *F*, *t*, *r*) with confidence intervals, effect sizes, degrees of freedom and *P* value noted<br>*Give P values as exact values whenever suitable.*
☒	☐	For Bayesian analysis, information on the choice of priors and Markov chain Monte Carlo settings
☒	☐	For hierarchical and complex designs, identification of the appropriate level for tests and full reporting of outcomes
☒	☐	Estimates of effect sizes (e.g. Cohen's *d*, Pearson's *r*), indicating how they were calculated

Our web collection on statistics for biologists contains articles on many of the points above.

Software and code

Policy information about availability of computer code

Data collection	Leica Stellaris 8 Falcon laser scanning confocal microscope, Tecnai-12 transmission electron microscope
Data analysis	GraphPad Prism (version 10.2.0 (392))<br>ImageJ (Fiji, version 1.54f)<br>IMOD software package (version 4.11)

For manuscripts utilizing custom algorithms or software that are central to the research but not yet described in published literature, software must be made available to editors and reviewers. We strongly encourage code deposition in a community repository (e.g. GitHub). See the Nature Portfolio guidelines for submitting code & software for further information.

Data

Policy information about availability of data

All manuscripts must include a data availability statement. This statement should provide the following information, where applicable:

- Accession codes, unique identifiers, or web links for publicly available datasets
- A description of any restrictions on data availability
- For clinical datasets or third party data, please ensure that the statement adheres to our policy

Source data are provided with this paper

Research involving human participants, their data, or biological material

Policy information about studies with human participants or human data. See also policy information about sex, gender (identity/presentation), and sexual orientation and race, ethnicity and racism.

Reporting on sex and gender	The study did not involve human participants
Reporting on race, ethnicity, or other socially relevant groupings	The study did not involve human participants
Population characteristics	The study did not involve human participants
Recruitment	The study did not involve human participants
Ethics oversight	The study did not involve human participants

Note that full information on the approval of the study protocol must also be provided in the manuscript.

Field-specific reporting

Please select the one below that is the best fit for your research. If you are not sure, read the appropriate sections before making your selection.

[x] Life sciences [ ] Behavioural & social sciences [ ] Ecological, evolutionary & environmental sciences

For a reference copy of the document with all sections, see nature.com/documents/nr-reporting-summary-flat.pdf

Life sciences study design

All studies must disclose on these points even when the disclosure is negative.

Sample size	Sample size was based on literature (Fu et al., 2012; Fu. et al., 2015; Kovacs et al., 2018).
Data exclusions	Any centriole imaged that was not fully in focus was excluded from analysis.
Replication	Each experiment was replicated independently more than three times, giving consistent results.
Randomization	Flies had to be allocated into experimental groups based on their genotype. Therefore, randomization was not relevant to our study.
Blinding	The phenotypes observed in our study were clear cut (and verified by a second person from the lab). Therefore, blinding was not relevant in our study.

Reporting for specific materials, systems and methods

We require information from authors about some types of materials, experimental systems and methods used in many studies. Here, indicate whether each material, system or method listed is relevant to your study. If you are not sure if a list item applies to your research, read the appropriate section before selecting a response.

Materials & experimental systems

n/a	Involved in the study	
[ ]	[x]	Antibodies
[x]	[ ]	Eukaryotic cell lines
[x]	[ ]	Palaeontology and archaeology
[ ]	[x]	Animals and other organisms
[x]	[ ]	Clinical data
[x]	[ ]	Dual use research of concern
[x]	[ ]	Plants

Methods

n/a	Involved in the study	
[x]	[ ]	ChIP-seq
[x]	[ ]	Flow cytometry
[x]	[ ]	MRI-based neuroimaging

Antibodies

Antibodies used	1. Rabbit anti-Asl (Drosophila): generated previously by the authors and described in Dzhindzhev et al, 2010; Dilution - 1:16,000<br>2. Chicken anti-dPLP: described in Rodriguez-Martins et al, 2007, obtained from the authors of that study; Dilution - 1:1000<br>3. Rabbit anti-Ana1: described in Fu et al, 2016, obtained from the authors of that study; Dilution - 1:5000<br>4. Guinea pig anti-Cep135: generated in the lab using Scottish National Blood Transfusion Service; Dilution - 1:500<br>5. Goat anti-chicken IgY Alexa Fluor 488 (Catalog # A-11039) Life Technologies; Dilution - 1:500

	6. Goat anti-guinea pig IgG Alexa Fluor 488 (Catalog # A-11073) Life Technologies; Dilution - 1:500<br>7. Goat anti-rabbit IgG Alexa Fluor 568 (Catalog # A-11011) Life Technologies; Dilution - 1:500<br>8. Goat anti-chicken IgY Alexa Fluor 647 (Catalog # A32933) Life Technologies; Dilution - 1:500
Validation	1. Rabbit anti-Asterless (Drosophila): Validated by western blot in Drosophila D-Mel2 cells and by immunofluorescence in Drosophila embryos<br>2. Chicken anti-dPLP: Validated by immunofluorescence in Drosophila S2 cells<br>3. Rabbit anti-Ana1: Validated by western blotting, immunofluorescence in D-Mel2 cells and Drosophila testes<br>4. Guinea pig anti-Cep135: Validated by western blotting, immunofluorescence in D-Mel2 cells

Animals and other research organisms

Policy information about studies involving animals; ARRIVE guidelines recommended for reporting animal research, and Sex and Gender in Research

Laboratory animals	Drosophila melanogaster (fruit fly), strain w[1118] and its derivatives, males, age - pharate adult stage
Wild animals	The study did not involve wild animals.
Reporting on sex	Findings apply to only male flies. The study focused on the effects of the mutation on the male germline cells.
Field-collected samples	This study did not involve samples collected from the field.
Ethics oversight	No ethical approval was required

Note that full information on the approval of the study protocol must also be provided in the manuscript.

Plants

Seed stocks	*Report on the source of all seed stocks or other plant material used. If applicable, state the seed stock centre and catalogue number. If plant specimens were collected from the field, describe the collection location, date and sampling procedures.*
Novel plant genotypes	*Describe the methods by which all novel plant genotypes were produced. This includes those generated by transgenic approaches, gene editing, chemical/radiation-based mutagenesis and hybridization. For transgenic lines, describe the transformation method, the number of independent lines analyzed and the generation upon which experiments were performed. For gene-edited lines, describe the editor used, the endogenous sequence targeted for editing, the targeting guide RNA sequence (if applicable) and how the editor was applied.*
Authentication	*Describe any authentication procedures for each seed stock used or novel genotype generated. Describe any experiments used to assess the effect of a mutation and, where applicable, how potential secondary effects (e.g. second site T-DNA insertions, mosiacism, off-target gene editing) were examined.*
